# Isolation of Lessertiosides A and B and Other Metabolites from *Lessertia frutescens* and Their Neuroprotection Activity

**DOI:** 10.3390/plants13213076

**Published:** 2024-11-01

**Authors:** Kadidiatou O. Ndjoubi, Sylvester I. Omoruyi, Robert C. Luckay, Ahmed A. Hussein

**Affiliations:** 1 Chemistry Department, Cape Peninsula University of Technology, Symphony Rd. Bellville, Cape Town 7535, South Africa; dickakadi@yahoo.fr; 2 School of Anatomical Sciences, Faculty of Health Sciences, University of the Witwatersrand, Parktown, Johannesburg 2193, South Africa; sylvester.omoruyi@wits.ac.za; 3 Department of Chemistry and Polymer Science, Stellenbosch University, Matieland, Stellenbosch 7602, South Africa; rcluckay@sun.ac.za

**Keywords:** *Lessertia frutescens*, terpenoids, lessertiosides A and B, SH-SY5Y cells, neurotoxicity, neuroprotection

## Abstract

*Lessertia frutescens* (synonym *Sutherlandia frutescens*) is an important South African medicinal plant used traditionally to treat different human pathologies and is considered an adaptogenic plant. This study sought to isolate compounds from the plant and determine their protective potentials using SH-SY5Y cells and MPP^+^ (1-methyl-4-phenylpyridinium) to mimic Parkinson’s disease. The phytochemical analysis of a 70% aqueous methanolic extract of *L. frutescens* leaves resulted in the isolation and identification of 11 pure compounds (**1**–**11**), among which compounds **1** and **2** were identified as new metabolites. The new compounds were characterised using IR, UV, NMR, and HRESIMS and were given the trivial names lessertiosides A (**1**) and B (**2**). Additionally, the flavonoids 8-methoxyvestitol (**7**) and mucronulatol (**8**) were isolated for the first time from the plant. The biological actions show that the isolated compounds had negligible toxicity on SH-SY5Y cells and improved cell viability in the cells exposed to MPP^+^. Furthermore, as a mechanism of action, the compounds could sustain cellular ATP generation and prevent MPP^+^-induced apoptotic cell death. Our findings provide evidence for the neuroprotective properties of compounds isolated from *L. frutescens* in MPP^+^-induced neuronal damage for the first time and create an avenue for these compounds to be further investigated to elucidate their molecular targets.

## 1. Introduction

Neuroprotection is associated with the capacity of treatment to mitigate neuronal cell death in short-term and long-term neurodegenerative conditions affecting the central nervous system [1,2]. Neurodegenerative diseases (ND) impact the function and composition of the entire nervous system [3], often leading to permanent brain damage. This damage limits various functions, including speech, mobility, bladder and bowel functions, and cognitive abilities, which is evident in Parkinson’s disease (PD) [4]. PD is a complicated neurological condition defined by the gradual and selective depletion of A9 midbrain dopaminergic (DA) neurons localised in the substantia nigra pars compacta (SNpc), accompanied by the collection of aggregated α-synuclein deposits known as Lewy bodies [3,5]. It ranks as the second most prevalent ND and affects about 1% of individuals between the ages of 65 and 70, with a higher prevalence of 4-5% among those over 80 [6]. By 2030, the number of PD patients is expected to surge by 30%, impacting not only the patients’ lifestyles but also the economy and society [7].

Although PD presents non-motor symptoms, clinical diagnosis typically involves observing motor symptoms, including bradykinesia, postural instability, resting tremors, and muscle rigidity [8]. Motor symptoms become evident at a later stage of the disease, often after patients have already lost approximately 80% of their striatal dopamine, equivalent to a 50% loss of DA neurons within the bulb, years before the SNpc of PD patients is affected [9,10,11]. The actual aetiology underscoring PD occurrence is not fully elucidated. However, age environment, genetics, iron accumulation, glutamate excitotoxicity, lipid peroxidation, lysosomal and mitochondrial dysfunction, neuroinflammation, nitrosative stress, oxidative stress, and proteasomal alterations contribute to its development [11,12,13,14]. While preclinical studies have identified potential therapies successful in PD animal models, translating these findings into successful clinical trials remains challenging [11].

At the moment, curative medication for PD is lacking. While the dopaminergic drug Levodopa effectively manages symptoms, its prolonged use often leads to adverse effects such as dyskinesia, drowsiness, low blood pressure, nausea, vomiting, and psychosis [15]. Thus, to combat neuronal cell loss and seek a cure for PD, researchers have been diligently searching for novel and efficient anti-Parkinson treatments, including plant-derived drugs. One promising area of research centers on *L. frutescens*, formerly known as *S. frutescens*, among others (*Colutea grandiflora*; *C. frutescens*, *C. frutescens*) [16]. This widely used South African medicinal plant is recognized for its potential therapeutic properties in treating various diseases such as diarrhoea, urinary tract infection, rheumatism, inflammation, intestinal pain, haemorrhoids, internal cancers, eye diseases, chickenpox, and skin disorders, as well as alleviating physical and mental stress [17,18,19]. While our previous work demonstrated the neuroprotective activity of *L. frutescens* extract [20], the current study evaluates the neuroprotective effects of compounds isolated from L. frutescens. These compounds were tested against the neurotoxin 1-methyl-4-phenylpyridinium (MPP^+^), which induces PD motor symptoms by limiting the function of mitochondrial complex I, which results in reduced cellular adenosine triphosphate (ATP) and augmentation of oxidative stress [21,22].

## 2. Results

### 2.1. Purification of L. frutescens Secondary Metabolites

**Figure 1:**
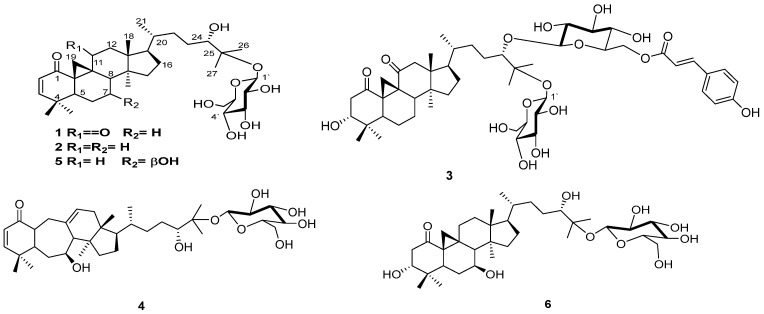

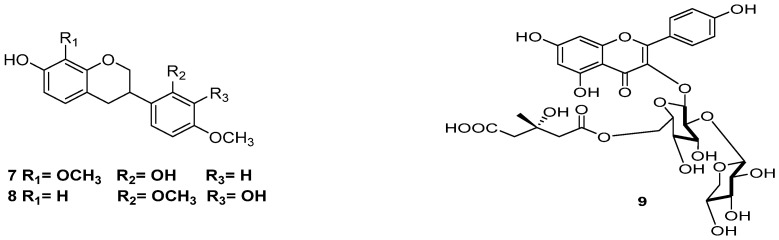
Chemical structure of *L. frutescens* isolated compounds (**1**–**9**).

Sequential silica gel 60, Sephadex LH-20 column chromatography, and semi-preparative HPLC were used to isolate eleven compounds (Figure 1) from the aqueous methanolic extract of *L. frutescens*. The structure elucidation of the new compounds (**1**–**2**) was accomplished by interrogating NMR spectroscopic data and complementary analyses such as IR and HR-ESIMS data. The known compound (**3**–**11**) structures were confirmed by comparing their NMR data with the reported ones. ^1^H and ^13^C NMR data of compounds **1** and **2** are provided in Table 1.

### 2.2. Dose–Response of L. frutescens Compounds

**Figure 2:**
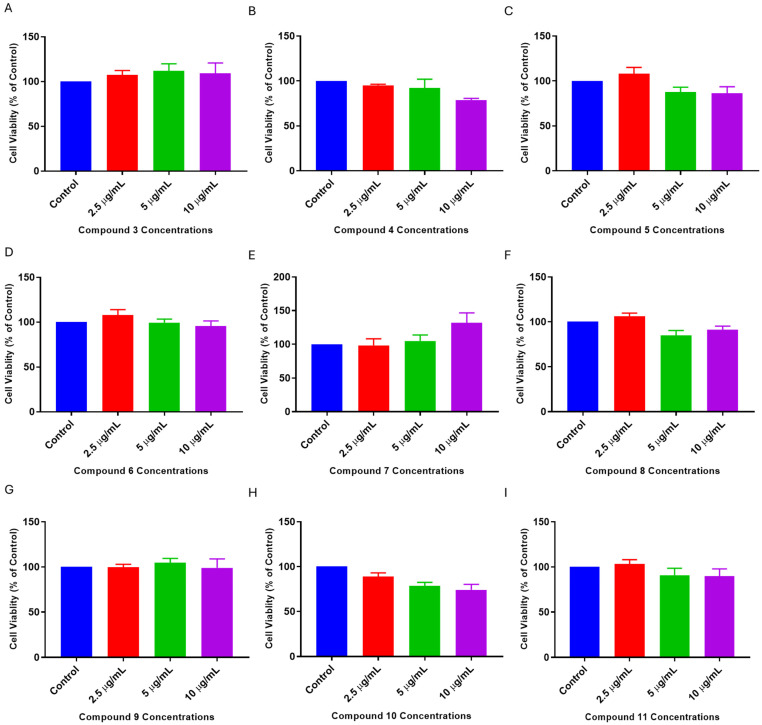
Cytotoxicity of *L. frutescens* compounds (**3**–**11**) on SH-SY5Y cells. SH-SY5Y cells were treated with concentrations of 2, 5, and 10 µg/mL of compounds for 24 h. After treatment, MTT assays were performed. The absorbance values obtained from quadruplicate wells were expressed as a percentage of the control. The bars in the graphs represent the means ± SEM of experiments performed in triplicates.

To determine the optimal concentrations of compounds that will not be cytotoxic and, in turn, show neuroprotection in the cells following neurotoxicity, the cells were treated with compounds numbered **3**–**11** at concentrations of 2.5, 5, and 10 µg/mL for 24 h, and an MTT cell viability assay was performed. The results showed that all compounds did not significantly induce cytotoxicity to the cells (Figure 2). Indeed, some compounds, including **4**, **5**, **6,** and **10**, had a concentration-dependent decrease in cell viability, with the 2.5 µg/mL concentration showing the highest viability. On the other hand, compound **7** had a concentration-dependent increase in cell viability. Altogether, these results indicate that the compounds showed minimal cytotoxicity effects on the SH-SY5Y cells under the current treatment conditions. Nevertheless, it should be acknowledged that 2.5 µg/mL had the least effect on the viability of cells for most of the compounds.

### 2.3. L. frutescens Compounds Protect SH-SY5Y Cells Following MPP^+^ Toxicity

**Figure 3:**
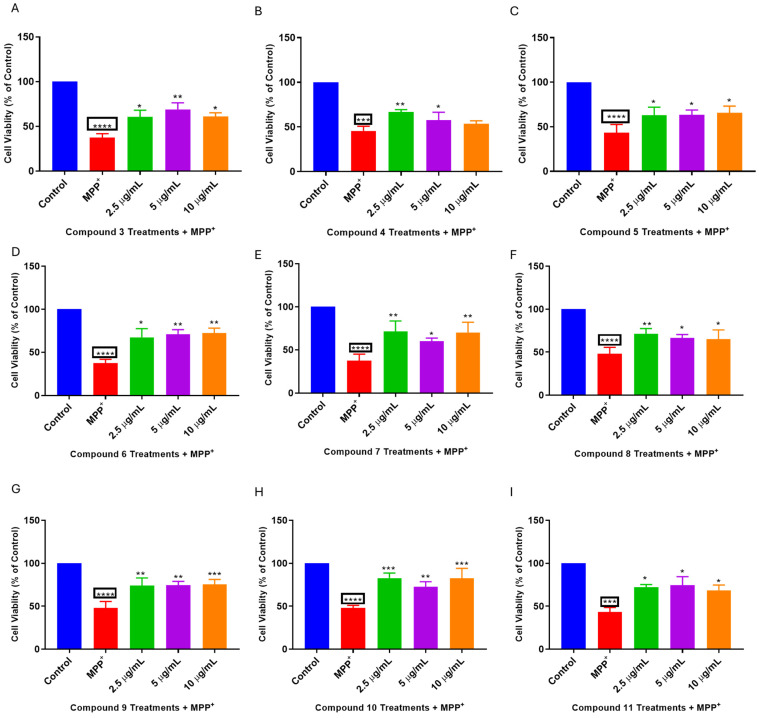
*L. frutescens* compounds (**3**–**11**) protect SH-SY5Y cells from MPP^+^ toxicity. SH-SY5Y cells were treated with 2, 5, and 10 µg/mL concentrations compounds for 2 h before the addition of MPP^+^ and cells were incubated for 24 h, followed by MTT assays. Absorbance values obtained from quadruplicate wells were calculated as a percentage of control. Bars represent the means ± SEM of three independent experiments, and the significance of difference indicated with * *p* < 0.05, ** *p* < 0.01, *** *p* < 0.001 and **** *p* < 0.0001 when cells pre-treated with compounds were compared to MPP^+^ and the boxed asterisks represent the comparison between MPP^+^ and the control cells.

Following optimisation of the concentration to be used for the neuroprotection studies, the neuroprotective potentials of the compounds (**3**–**11**) were evaluated in the SH-SY5Y cells co-treated with compounds and MPP^+^. Figure 3 confirms that under the concentrations utilised, all compounds significantly increased the viability of SH-SY5Y cells following MPP^+^ toxicity except for the cells treated with compound **4** (Figure 3) at the 10 µg/mL concentration. Indeed, MPP^+^ reduced cell viability to about 37 to 48%, while treatment with concentrations of the compounds rescued the cells from MPP^+^ toxicity. Since the 2.5 µg/mL concentration for the majority of the compounds showed the most increased cell viability, this concentration was selected for further evaluation of the neuroprotective effects of the compounds.

### 2.4. Effects of L. frutescens Compounds in ATP Production Following MPP^+^ Toxicity

**Figure 4:**
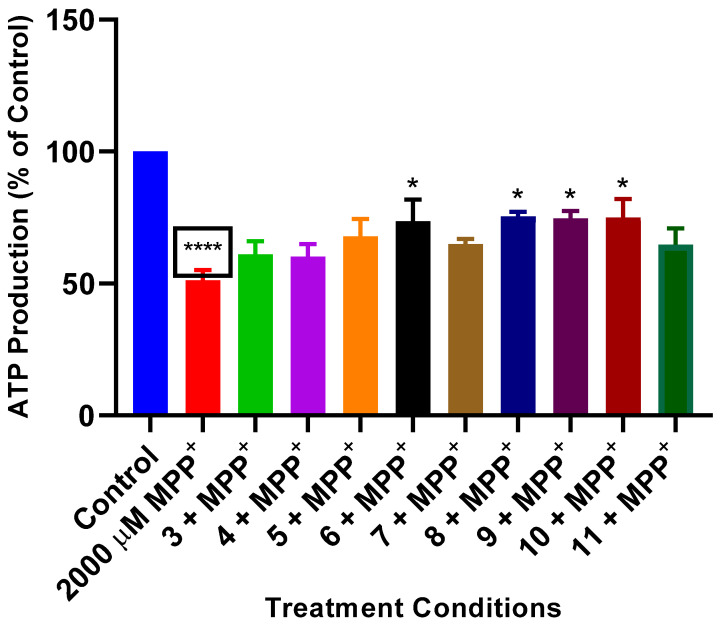
Impact of *L. frutescens* compounds (**3**–**11**) on ATP production. SH-SY5Y cells were treated with 2, 5, and 10 µg/mL concentrations compounds for 2 h before the addition of MPP^+^ and cells were incubated for 24 h, followed by ATP assays. Luminescence values obtained were expressed as a percentage of the control. Bars represent means ± SEM of three independent experiments, and the significance of difference is indicated with * *p* < 0.05 and **** *p* < 0.0001 when cells pre-treated with compounds were compared to MPP^+^, and the boxed asterisks represent the comparison between MPP^+^ and the control cells.

To further assess the neuroprotective potentials of compounds **3**–**11** isolated from *L*. *frutescens*, their impact on ATP production was determined in SH-SY5Y cells pre-treated with compounds before exposure to MPP^+^. The results highlight that whereas MPP^+^ significantly inhibited ATP production, pre-treatment with compounds showed an increase in ATP production (Figure 4). However, this increase was only significant for the cells treated with compounds **6**, **8**, **9,** and **10**. Indeed, MPP^+^ reduced ATP production to about 51%, but pre-treatment with compounds **6**, **8**, **9,** and **10** increased ATP production to 73, 75, 74, and 75%, respectively. Altogether, these results suggest that the prevention of ATP degeneration induced by MPP^+^ is involved in the neuroprotection mechanisms of compounds isolated from *L. frutescens*.

### 2.5. L. frutescens Compounds Rescue Cells from MPP^+^-Induced Apoptosis

**Figure 5:**
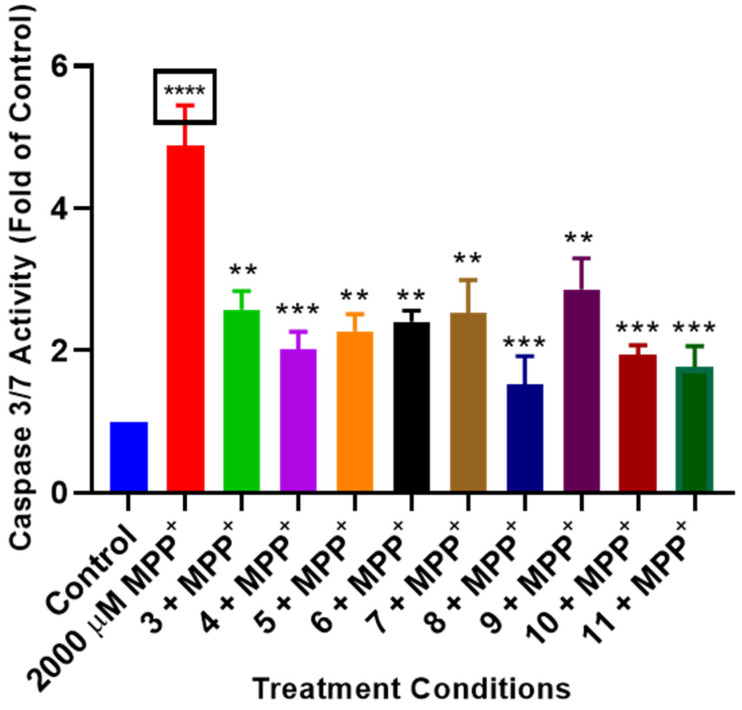
Impact of *L. frutescens* compounds (**3**–**11**) on ATP production. SH-SY5Y cells were treated with 2, 5, and 10 µg/mL concentrations compounds for 2 h before the addition of MPP^+^ and cells were incubated for 24 h, followed by caspase 3/7 Glo assays. Luminescence values obtained were represented as fold of control. Bars represent means ± SEM of three independent experiments, and the significance of the difference is indicated with ** *p* < 0.01, *** *p* < 0.001, and **** *p* < 0.0001 when cells pre-treated with compounds were compared to MPP^+^, and the boxed asterisks represent the comparison between MPP^+^ and the control cells.

Apoptosis is a mechanism of cell death, and caspase 3/7 activities in the cells can indicate apoptosis. Thus, to investigate apoptosis in the cells, the activities of caspase 3/7 were measured using the Promega caspase assay kit in cells pre-treated with compounds before exposure to MPP^+^. The results showed that compounds **3**–**11** significantly mitigated the apoptosis-inducing effects of MPP^+^ by attenuating the elevated levels of caspase 3/7 activities in the cells. When caspase 3/7 activities were expressed as a fold of the control, which control cells set to 1, MPP^+^ increased caspase 3/7 activities to approximately 5-fold of the control while the compounds ranged from 1.5- to 2.8-fold of the control (Figure 5). It is important to highlight that compound **8** inhibited apoptosis the most to about 1.5-fold of the control. These results suggest that the studied compounds might carry out their neuroprotective effects by mitigating MPP^+^-induced apoptosis in the cells.

## 3. Discussion

The chromatographic purification of a methanolic extract from *L. frutescens* yielded eleven compounds (**1**–**11**). Compounds **3** and **4** were recently reported as sutherlandiosides K and E, respectively [23]. However, the trivial name sutherlandioside E was already assigned to the 9,10-seco-cycloartane-type diglycoside bearing an unprecedented 5/7/6/5 ring skeleton isolated earlier by Fu et al. (2012) [24]. Compound **7** was identified as 8-methoxyvestitol and previously isolated from *Astragalus trigonus* [25], while compound **8** was identified as mucronulatol and previously isolated from *Machaerium mucronularum* [26], both **7** and **8** were isolated for the first time from *L. frutescens*. On the other hand, compound **6** (sutherlandioside B), compound **9** (sutherlandin C), compound **10** (proline), and compound **11** (D-pinitol) are well-known *L. frutescens* compounds.

The neuroprotective effects of these compounds were evaluated against MPP^+^-induced toxicity in SH-SY5Y cells. The study investigated ATP production and caspase 3/7 activity, which are critical indicators of cell viability and apoptotic pathways, respectively. Our findings suggest that the *L. frutescens* compounds confer neuroprotection against MPP^+^-induced neurotoxicity in SH-SY5Y cells, a model often used to mimic PD pathology.

### 3.1. Structure Elucidation

Compound **1** (lessertioside A), HR-ESIMS exhibited a molecular ion peak of 633.39 *m*/*z* corresponding to a molecular formula of C_36_H_56_O_9_. The IR absorptions display characteristic bands at 3354, 2959, 1660, 1457, and 1143 cm^−1^, revealing the presence of alkoxyl, carbonyl, hydroxyl, alkyl, and olefin groups. The ^1^H NMR showed the signals of six tertiary methyl groups at δ_H_ 0.74 (*s*, Me-18), 1.14 (*s*, Me-26), 1.17 (*s*, Me-27), 1.08 (*s*, Me-28), 1.02 (*s*, Me-29), and 1.10 (*s*, Me-30) as well as one secondary methyl group at δ_H_ 0.86 (*d*, *J* = 6.1 Hz, Me-21). In addition, an isolated methylene group was observed at δ_H_ 0.93 (H-19α) and 1.79 (H-19β) as well as resonances of seven methylene groups at δ_H_ 1.41 (H-6α), 1.59 (H-6β), 1.33 (H-7α), 1.57 (H-7β), 2.52 (*d*, *J* = 15.2 Hz, H-12α), 2.78 (*d*, *J* = 15.2 Hz, H-12β), 1.36 (H-15α), 1.41 (H-15β), 1.41 (H-16), 1.40 (H-22α), 1.92 (*m*, H-22β), 1.08 (H-23α), and 1.57 (H-23β). The spectrum also showed a low field proton attached to an oxygenated carbon at δ_H_ 3.31 (*t*, *J* = 7.0 Hz, H-24); two olefinic protons at δ_H_ 5.72 (*d*, 10.3, H-2) and 6.62 (*d*, 10.3, H-3) as well as the signals of three methine groups 2.14 (*dd*, *J* = 12.5; 4.3 Hz, H-5), 2.25 (*t*, *J* = 7.9 Hz, H-8), and 1.36 (H-20) revealing the aglycon to be a cycloartane-type triterpenoid. The ^1^H NMR also displayed a signal of an anomeric proton at δ_H_ 4.46 (*d*, *J* = 8.2 Hz, H-1′) and a cluster of protons ranging from δ_H_ 3.10 to 3.76, indicating the presence of a glucose unit.

The ^13^C NMR signals showed 36 peaks which were categorised according to DEPT-135 into seven methyls at δ_C_ 16.6 (C-18), 19.0 (C-21), 21.5 (C-26), 23.9 (C-27), 28.7 (C-28), 21.5 (C-29), 19.1 (C-30); nine methylene at δ_C_ 22.6 (C-6), 19.5 (C-7), 53.2 (C-12), 34.2 (C-15), 22.8(C-16), 34.9 (C-19), 28.8 (C-22), 29.3 (C-23), 62.7 (C-6′); thirteen methines at δ_C_ 126.8 (C-2), 161.8 (C-3), 45.7 (C-5), 39.3 (C-8), 52.2 (C-17), 37.8 (C-20), 79.5 (C-24), 98.6 (C-1′), 75.2 (C-2′), 78.1 (C-3′), 71.1 (C-4′), 77.7 (C-5′); and seven quaternary carbons at δ_C_ 199.8 (C-1), 39.2 (C-4), 36.2 (C-9), 39.5 (C-10), 212.6 (C-11), 48.7 (C-13), 50.6 (C-14), 82.0 (C-25).

**Figure 6:**
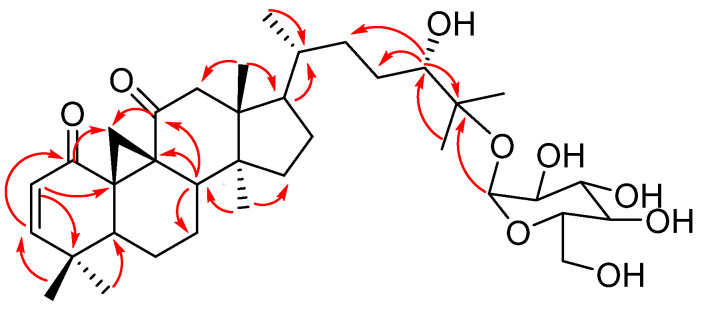
HMBC correlations of compound **1** (H→C).

Based on the HMBC spectrum, the correlation (Figure 6) between the anomeric proton H-1′ and C-25 further confirms the attachment of the glucose group to C-25. It was also observed that the hydroxymethine moiety (C-24) correlated with the methyl groups at C-26 and C-27 as well as the quaternary carbon C-25, which in turn was linked to H-23, H-22 in the COSY spectrum. Moreover, the chemical shift of C-24, located within the range typical for *S* stereoisomers (77.1 to 78.6 ppm), suggests that C-24 is in the *S* configuration [23,27,28]. The chemical shifts observed for the side chain (C-20 to C-27) attached to C-17 were similar to those reported for sutherlandioside A-D. The ^1^H-^1^H COSY and HMBC spectra indicated correlations between H-5, H-6, and H-8 with the methylene signal at C-7. HMBC correlations from H-28/H-29 to C-3, H-3 to C-1, and H-2 to C-4 indicated the presence of an olefinic bond between C-2 and C-3. These findings suggest a structural resemblance between sutherlandioside D (compound **5**) and compound **1** [28]. The glucose unit was confirmed as β-configuration according to the coupling constants of the anomeric proton (*J* = 8.2 Hz). The chemical shifts of the protons and the carbons were compared carefully with similar compounds isolated from the same source [23,24,27,28].

However, unlike sutherlandioside D, compound **1** does not have a hydroxymethine at C-7 but a methylene group. Additionally, an extra carbonyl group at C-11 was found to be linked to the protons at position 19. The chemical shifts of H-12 were detected in the lower field compared to the resonance of H-12 at δ_H_ 1.62 (H-12α) and δ_H_ 1.35 (H-12β) in compound 5, further confirming the second carbonyl group to be attached to C-11. A carbonyl group at position 11 indicates that compound **1** is also similar to sutherlandioside C [28]. However, unlike sutherlandioside C, compound **1** features a double bond between C-2 and C-3 instead of a hydroxyl group at C-3. Following the data from HRESIMS, IR, and NMR analyses, compound 1 has been characterised as 24*S*,25-dihydroxycycloart-2-en-1,11-dione-25-*O*-β-D-glucopyranoside (lessertioside A).

Furthermore, compound **2** (lessertioside B) HR-ESIMS displayed a molecular ion peak of 619.41 *m*/*z*. The molecular formula of compound **2** was deduced as C_36_H_58_O_8_, which corroborated with the HR-ESIMS and The IR absorption bands at 3292, 2938, 1674, 1457, and 1209, 3292 cm^−1^, showing the presence of hydroxyl, olefin, and carbonyl groups. The ^1^H NMR showed the signals of seven methyl groups at δ_H_ 0.87 (*s*, Me-18), 0.81 (*d*, *J* = 6.7 Hz, Me-21), 1.10 (*s*, Me-26), 1.13 (*s*, Me-27), 1.02 (*s*, Me-28), 0.89 (*s*, Me-29) and 0.88 (*s*, Me-30). Additionally, the resonance of nine methylene groups at δ_H_ 1.20 (H-6α), 1.41 (H-6β), 1.24 (H-7α), 1.84 (H-7β), 2.09 (H-11α), 2.25 (H-11β), 1.23 (H-12α), 1.52 (H-12β), 1.21 (H-15α), 1.51 (H-15β), 1.60 (H-16), 1.24 (H-19α), 0.95 (*d*, *J* = 4.5 Hz, H-19β), 1.74 (H-22α), 2.24 (H-22β), 1.02 (H-23α), and 1.52 (H-23β) were observed. The ^1^H NMR spectrum also showed six methine groups at δ_H_ 5.69 (*d*, *J* = 10.1 Hz, H-2), 6.61 (*d*, *J* = 10.1 Hz, H-3), 2.18 (*dd*, *J* = 12.8; 4.5 Hz, H-5), 1.81 (H-8), 1.30 (H-20), and 3.27 (*d*, *J*= 7.4 Hz, H-24). The presence of a glucose unit was confirmed by the anomeric proton signal at δ_H_ 4.40 (d, *J* = 7.4 Hz, H-1′) and the cluster of protons at δ_H_ 3.16 (*t*, *J* = 8.5 Hz, H-2′), 3.20 (H-3′), 3.18 (H-4′), 3.17 (H-5′), 3.59 (*dd*, *J* = 12.3; 4.3 Hz, H-6α′), and 3.71 (*d*, *J* = 12.3 Hz, H-6β′).

The ^13^C NMR signals showed 36 peaks; 30 were attributed to the triterpenoid nucleus, while the remaining 6 displayed a glucose unit. These peaks were categorised according to DEPT-135 and HSQC into seven methyls at δ_C_ 18.2 (C-18), 19.4 (C-21), 21.6 (C-26), 24.2 (C-27), 28.4 (C-28), 21.4 (C-29), 19.7 (C-30); nine methylene at δ_C_ 25.1 (C-6), 26.8 (C-11), 34.5 (C-12), 36.4 (C-15), 22.0 (C-16), 34.7 (C-19), 35.3 (C-22), 29.7 (C-23), 62.9 (C-6′); thirteen methines at δC 129.5 (C-2), 161.5 (C-3), 46.2 (C-5), 29.2 (C-7), 48.0 (C-8), 54.0 (C-17), 38.1 (C-20), 79.8 (C-24), 98.8 (C-1′), 75.4 (C-2′), 78.3 (C-3′), 71.8 (C-4′), 77.9 (C-5′); and seven quaternary carbons at δ_C_ 204.3 (C-1), 38.8 (C-4), 31.8 (C-9), 36.9 (C-10), 46.4 (C-13), 50.8 (C-14), 82.2 (C-25).

**Figure 7:**
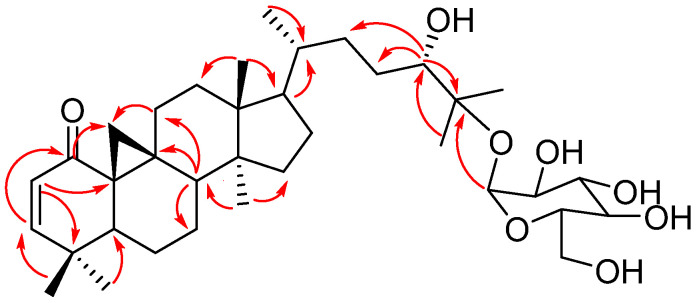
HMBC correlations of lessertioside B (H→C).

The chemical shifts of the glucose unit are identical to compound **1**. According to the ^1^H and ^13^C NMR spectra, compounds **2** and **1** exhibit striking structural similarities with one notable distinction: the absence of a carbonyl group at C-11. This discrepancy has been thoroughly corroborated by the HMBC (Figure 7) and COSY spectra, which showed the correlation between H-19 and H-11, which in turn was connected to C-11 in the HSQC spectrum, indicating the carbonyl group at C-11 in compound **1** has been reduced to a methylene group in compound **2**. Thus, the structure of compound **2** was characterised as 24*S*,25-dihydroxycycloart-2-en-1-one-25-*O*-β-D-glucopyranoside (lessertioside B).

### 3.2. Neuroprotective Effects of the Isolated Compounds

Their impact on cellular ATP was investigated to elucidate the mechanism of action of the compounds. ATP is the primary energy currency of the cell, and its depletion is a hallmark of mitochondrial dysfunction, which is closely linked to neurodegenerative diseases such as PD [29,30]. MPP^+^ causes a significant decrease in ATP levels by blocking complex I of the mitochondrial electron transport chain, resulting in reduced cellular energy and subsequent cell death [31,32,33]. In this study, treatment with *L. frutescens* compounds (**3**–**11**) resulted in substantial preservation of ATP levels in SH-SY5Y cells exposed to MPP^+^. This suggests that these compounds may mitigate mitochondrial dysfunction, potentially through direct interaction with mitochondrial components or by scavenging free radicals that exacerbate oxidative stress and energy failure. Furthermore, the maintenance of ATP levels in the presence of MPP^+^ implies that *L. frutescens* compounds help sustain cellular energy metabolism, which is crucial for neuronal survival [34,35]. This effect could be attributed to the enhancement of mitochondrial biogenesis, protection of mitochondrial integrity, or activation of alternative energy-generating pathways. Further investigation is warranted into the precise mechanisms by which *L. frutescens* compounds may impact ATP levels following MPP^+^ toxicity.

Following the depletion of ATP in cells, cells may proceed to cell death [36,37]. Caspase 3/7 are central executors of apoptosis, activated during the terminal stages of the intrinsic/extrinsic apoptotic pathway, often triggered by mitochondrial dysfunction and energy deficits [38,39]. MPP^+^ exposure triggered increased levels of caspase 3/7 activities, consistent with the induction of apoptosis in SH-SY5Y cells [40,41]. However, pre-treatment with *L. frutescens* compounds significantly reduced caspase 3/7 activation, indicating an anti-apoptotic effect. The inhibition of caspase 3/7 activities suggests that *L. frutescens* compounds may interfere with upstream events leading to caspase activation, such as cytochrome c release, apoptosome formation, or other pro-apoptotic signalling pathways [39,42]. By preventing the activation of these caspases, *L. frutescens* compounds likely help preserve cellular integrity and function, thereby offering a protective effect against MPP^+^-induced neurotoxicity. In conjunction with preserving ATP levels, this anti-apoptotic action highlights the potential of *L. frutescens* compounds in maintaining neuronal viability in the face of mitochondrial stress.

## 4. Materials and Methods

### 4.1. Chemicals, Materials, and Reagents

The leaves powder of *L. frutescens* was donated by Afriplex (Paarl, South Africa) in 2018. The Silica gel 60 (0.063–0.200 mm), Sephadex (LH-20), and aluminium thin layer chromatography plate supplied by Merck (Cape Town, South Africa) were used. Hexane, ethyl acetate, and methanol AR grades were purchased from the local market (Kimix, Cape Town, South Africa). The Shimadzu LC-20 HPLC system has a quaternary pump (LC-20AD), a diode array detector (DAD, SPD-M20A), and a manual injector. HPLC grade methanol and a reversed-phase C18 column (25 × 1 cm, 5 μm, Supelco, Merck, South Africa) were used for purification. The detector was operating at wavelengths 254, 272, and 366 nm. The 1D NMR (^1^H, ^13^C, and DEPT-135) and 2D NMR (HMBC, HSQC, and COSY) spectra were measured using a Bruker spectrometer (Rheinstetten, Germany) operating at 400/100 MHz for ^1^H and ^13^C NMR, respectively.

### 4.2. Extraction of Plant Sample

The ground plant material (1.0 kg) underwent maceration in 70% aqueous methanol for 24 h at room temperature, followed by filtration. The process was repeated twice. The combined filtrates were evaporated using a Rotavapor, producing 64.6 g of total extract.

### 4.3. Isolation of L. frutescens Secondary Metabolites

The purification and isolation of secondary metabolites from the aqueous methanol extract were carried out using a combination of Sephadex LH-20 column chromatography, silica gel 60 column chromatography, and semi-preparative HLPC. All semi-preparative HPLC was conducted using HPLC grade methanol and deionised water as eluent at a flow rate of 1.0 mL/min, following a gradient of 60% to 80% methanol over 45 min, and 80% to 100% methanol for 15 min.

The total extract was fractionated using silica gel column chromatography. A gradient of hexane/ethyl acetate, followed by ethyl acetate/methanol with increasing polarity, was used, yielding twenty-seven fractions (I–XXVII), of which only fractions IX, X, XI, XII, XV, XVII, XIX, XX, XXII, and XXV were subjected to further purification. The remaining fractions were not considered due to their similarities with the selected fractions, except for fractions I to VII, which mainly contained fatty acids and chlorophyll components.

Fractions IX to XII were combined (4.2 g) and subjected to isocratic silica gel column chromatography using hexane: ethyl acetate (90:10), followed by Sephadex LH-20 column chromatography using 40% aqueous methanol. This purification process resulted in isolating compounds **7** (45.4 mg) and **8** (4.1 mg).

Fraction XV (500 mg) was subjected to Sephadex LH-20 column chromatography using 20% aqueous methanol, followed by semi-preparative HPLC, leading to the isolation of compounds **4** (5.9 mg) and **2** (4.1 mg) at retention times (R_t_) of 36 min and 62 min, respectively. Fraction XVII (1.0 g) underwent the same conditions to yield compounds **3** (R_t_ 35 min, 6.7 mg), **5** (R_t_ 36 min, 12.1 mg), and **6** (R_t_ 32.5 min, 8.9 mg).

Fractions XIX and XX were combined and subjected to silica gel column chromatography, followed by semi-preparative HPLC, resulting in the isolation of compound **1** (R_t_ 47.5 min, 9.8 mg). Fraction XXII (250 mg) was subjected to Sephadex LH-20 column chromatography using 30% aqueous methanol, followed by semi-preparative HPLC, leading to the isolation of compound **9** (R_t_ 29 min, 8.2 mg). Fraction XXV (500 mg) was chromatographed on Sephadex LH-20 using 30% aqueous methanol, resulting in the isolation of compounds **10** (43.3 mg) and **11** (17.8 mg).

### 4.4. Spectroscopic Data of Lessertiosides A and B

**Lessertioside A** (C_36_H_56_O_9_): colourless powder, ${\left[\alpha \right]}_{25}^{D}$ = −2.51 (*c* 0.3, MeOH); IR: 3354 cm^−1^ (OH), 2959 cm^−1^ (CH), 1660 cm^−1^ (C=O), 1457 cm^−1^ (C=C), 1143 cm^−1^ (C-O); UV: 316 nm; HRESIMS: *m*/*z* 633.3991 [M+H]^+^ (calculated for C_36_H_57_O_9_, 633.3997); ^1^H NMR (CDCl_3_) and ^13^C NMR (CDCl_3_): see Table 1. (See Supplementary Information Figures S1.1–S1.5).

**Lessertioside B** (C_36_H_58_O_8_): colourless powder, ${\left[\alpha \right]}_{25}^{D}$ = +3.06 (*c* 0.4, MeOH); IR: 3292 cm^−1^ (OH), 2938 cm^−1^ (CH), 1674 cm^−1^ (C=O), 1457 cm^−1^ (C=C), 1209 cm^−1^ (C-O); UV: 320 nm; HRESIMS: *m*/*z* 619.4190; [M+H]^+^ (calculated for C_36_H_59_O_8_, 619.4204); ^1^H NMR (CDCl_3_) and ^13^C NMR (CDCl_3_): see Table 1. (See Supplementary Information Figures S2.1–S2.5).

### 4.5. Cell Culture and Maintenance

The human neuroblastoma SH-SY5Y cells were generously provided by the Blackburn Laboratory at the University of Cape Town. The SH-SY5Y cells were chosen for this study as they are widely used in neuroscience research to model neurodegenerative diseases, including PD, due to their dopaminergic characteristics. The cells exhibit functional dopamine receptors and neurotransmitter uptake, mimicking the dopaminergic system’s physiology. The cells were cultured in Dulbecco’s Modified Eagle’s Medium (DMEM, Gibco, Life Technologies Corporation, Paisley, UK), with 10% fetal bovine serum (FBS, Gibco, Life Technologies Corporation, Paisley, UK), 100 U/mL penicillin, and 100 µg/mL streptomycin (Lonza Group Ltd., Verviers, Belgium) as supplements. Cultures were maintained at 37 °C in a humidified incubator with 5% CO_2_, with the cell growth medium being routinely replaced every three days. When the cells reached 70–80% confluency, they were sub-cultured using a 0.25% trypsin EDTA solution (Lonza Group Ltd., Verviers, Belgium).

### 4.6. Treatments

A 10 mg/mL stock solution of the individual compounds (**3**–**11**) was made in dimethyl sulfoxide (DMSO) (Sigma-Aldrich, St Louis, MO, USA). These solutions were diluted further in the growth medium to achieve the desired final concentrations. To optimise the concentration of compounds that will be neuroprotective, 10,000 cells were grown/well in the 96-well plate, followed by treatment with 2.5, 5, and 10 µg/mL concentrations. Control cells were treated with the same amount of DMSO as contained in the 10 µg/mL concentration of the compounds, and treatments lasted for 24 h. To establish neurotoxicity, 2000 µM MPP^+^ was used, consistent with our previous research [22]. For neuroprotective studies, the cells were similarly grown as mentioned above and pre-treated with the 2.5, 5, and 10 µg/mL concentrations of compounds for 2 h, followed by adding 2000 µM MPP^+^. The treatments were left for 24 h, and the vehicle-treated cells were used as controls.

### 4.7. Cell Viability Assay

The MTT [3-(4,5-dimethylthiazol-2-yl)-2,5-diphenyltetrazolium bromide] assay (Sigma-Aldrich, St Louis, MO, USA) was utilised to assess cell viability following treatment with either compound or after pre-treatment with compounds followed by MPP^+^. After treatment of cells as mentioned above, 10 or 20 µL (depending on well volume) of a 5 mg/mL MTT solution in PBS (Lonza Group Ltd., Verviers, Belgium) was added to each well and kept in the incubator for an additional 4 h at 37 °C. After the incubation period, the medium with the MTT dye was removed, and the MTT formazan was dissolved using 100 µL of DMSO. The absorbance was then read at 570 nm in a spectrophotometer (BMG Labtech Omega^®^ POLARStar, Ortenberg, Germany) and the percentage of viable cells was determined with the control cells as reference.

### 4.8. Adenosine Triphosphate Levels (ATP) Assay

The Mitochondrial ToxGlo ATP assay kit (Promega, Madison, WI, USA) was utilised to determine ATP production in the cells. Summarily, the cells were plated in a white-walled 96-well plate, and treatment was similar to what was previously outlined for the neuroprotection studies. Post-treatment, cells were processed per the manufacturer’s instructions by adding an equal volume of ATP detection reagent to each well and incubating at 37 °C for 30 min. After incubation, luminescence intensity was measured using the BMG Labtech Omega^®^ POLARStar multimode microplate reader, and the luminescence values were expressed as percentages of the control.

### 4.9. Caspase 3/7 Apoptosis Assay

To investigate cell apoptosis, the Caspase 3/7 assay kit (Promega, Madison, WI, USA) was used to measure caspase 3/7 activity following the manufacturer’s instructions. Briefly, cells were seeded in a white 96-well plate at a density of 10,000 cells per well and allowed to attach overnight. Subsequently, cells were pre-treated with compounds before adding 2000 µM MPP^+^. After 24 h of treatment, equal volumes of the caspase 3/7 assay mix were added to each well, and the luminescence intensity was measured using the BMG Labtech Omega^®^ POLARStar multimode microplate reader. The luminescent values of the treated cells were expressed as fold changes compared to the control.

### 4.10. Statistical Analysis

Graph Pad Prism Version 8 was used to analyse the data obtained from three independent experiments. The data were represented as means ± SEM (standard error of means), and a one-way analysis of variance (ANOVA) was used to analyse the differences between the treated cells and vehicle control cells, and a *p*-value < 0.05 was considered significant.

## 5. Conclusions

The discovery of new cycloartane glycosides highlights the significance of this plant as a source of bioactive compounds. It supports its widespread use in treating various human diseases in Southern Africa. The neuroprotective effects observed in this study underscore the potential of *L. frutescens* compounds as potential therapeutic agents in neurodegenerative diseases that warrant further investigation. By preserving ATP levels and inhibiting caspase 3/7 activation, these compounds could help counteract the energy deficits and apoptotic processes contributing to neurodegeneration. In the future, the molecular targets of the compounds and more investigation into the mechanisms of action should be carried out. In addition, in vivo studies and clinical trials will be necessary to determine the efficacy and safety of these compounds in a physiological context.

## Figures and Tables

**Table 1:** ^1^H and ^13^C NMR data of compounds **1** and **2**.

	1		2	
No	^13^C	^1^H, *muti*. (*J* = Hz)	^13^C	^1^H, *multi*. (*J* = Hz)
1	199.8		204.3	
2	126.8	5.72, *d* (10.3)	129.5	5.69, *d* (10.1)
3	161.8	6.62, *d* (10.3)	161.5	6.61, *d* (10.1)
4	39.2		38.8	
5	45.7	2.14, *dd* (12.5, 4.3)	46.2	2.18, *dd* (12.8, 4.5)
6	22.6	1.59 *; 1.41	25.1	1.41 *; 1.20 *
7	19.5	1.57 *; 1.33 *	29.2	1.84 *; 1.24 *
8	39.3	2.25, *t* (7.9)	48.0	1.81 *
9	36.2		31.8	
10	39.5		36.9	
11	212.6		26.8	2.25 *; 2.09 *
12	53.2	2.78, *d* (15.2) 2.52, d (15.2)	34.5	1.52 * 1.23 *
13	45.0		46.4	
14	50.6		50.8	
15	34.2	1.41 *; 1.36 *	36.4	1.51 *; 1.21 *
16	22.8	1.41 *	22.0	1.60 *
17	52.2	1.79, *m*	54.0	1.51 *
18	16.6	0.74, *s*	18.2	0.87, *s*
19	34.9	1.79 *; 0.93 *	34.7	1.24 *; 0.95, *d* (4.5)
20	37.8	1.36 *	38.1	1.30 *
21	19.0	0.86, *d* (6.1)	19.4	0.81, *d* (6.7)
22	28.8	1.92, *m*; 1.40 *	35.3	2.24 *; 1.74 *
23	29.3	1.57 *; 1.08 *	29.7	1.52 *; 1.02 *
24	79.5	3.31, *t* (7.0)	79.8	3.27, *d* (7.4)
25	82.0		82.2	
26	21.5	1.14, *s*	21.6	1.10, s
27	23.9	1.17, *s*	24.2	1.13, *s*
28	28.7	1.08, *s*	28.4	1.02, *s*
29	21.5	1.02, *s*	21.4	0.89, *s*
30	19.1	1.10, s	19.7	0.88, *s*
1′	98.6	4.46, *d* (8.2)	98.8	4.40, *d* (7.4)
2′	75.2	3.10, *t* (8.2)	75.4	3.16, *t* (8.5)
3′	78.1	3.30 *	78.3	3.20 *
4′	71.1	3.22 *	71.8	3.18 *
5′	77.7	3.21 *	77.9	3.17 *
6′	62.7	3.58, *dd* (11.1, 4.5) 3.76, *d* (11.1)	62.9	3.59, *dd* (12.3, 4.3) 3.71, *d* (12.3)

* Partially or fully overlapped signals.

## Data Availability

The raw data are available at https://www.esango.cput.ac.za (accessed on 1 October 2024).

## Supplementary Materials

The following supporting information can be downloaded at: https://www.mdpi.com/article/10.3390/plants13213076/s1, Figure S1.1. ^1^H NMR of compound **1**. Figure S1.2. ^13^C NMR of compound **1**. Figure S1.3. DEPT-135 of compound **1**. Figure S1.4. HSQC of compound **1**. Figure S1.5. HMBC of compound **1**. Figure S2.1. ^1^H NMR of compound **2**. Figure S2.2. ^13^C NMR of compound **2**. Figure S2.3. DEPT-135 of compound **2**. Figure S2.4. HSQC of compound **2**. Figure S2.5. HMBC of compound **2**.
