# Supplementary material for: Isolation of Lessertiosides A and B and Other Metabolites from Lessertia frutescens and Their Neuroprotection Activity
Source: Plants (Basel). 2024 Nov 1;13(21):3076. doi: 10.3390/plants13213076 (PMC11548272; doi:10.3390/plants13213076)
Supplement: Supplementary file 1 [file plants-13-03076-s001.zip › plants-3280784-supplementary.pdf]

Supplementary information

Isolation of Lessertiosides A and B and other Metabolites from *Lessertia frutescens* and Their Neuroprotection Activity.

Kadidiatou O. Ndjoubi[1], Sylvester I. Omoruyi[2], Robert C. Luckay[3] and Ahmed A. Hussein[1*]

[1] Chemistry Department, Cape Peninsula University of Technology, Symphony Rd. Bellville, Cape Town 7535, South Africa; dickakadi@yahoo.fr (K.O.N); mohammedam@cput.ac.za (A.A.H)
[2] School of Anatomical Sciences, Faculty of Health Sciences, University of the Witwatersrand, Parktown, Johannesburg 2193, South Africa; sylvester.omoruyi@wits.ac.za
[3] Department of Chemistry and Polymer Science, Stellenbosch University, Matieland, Stellenbosch 7602, South Africa; rcluckay@sun.ac.za
* Correspondence: mohammedam@cput.ac.za

NMR data of Compounds 1 and 2.:

1- Figure S1.1. ^{1}H NMR of compound **1.**
2- Figure S1.2. ^{13}C NMR of compound **1.**
3- Figure S1.3. DEPT-135 of compound **1.**
4- Figure S1.4. HSQC of compound **1.**
5- Figure S1.5. HMBC of compound **1.**
6- Figure S2.1. ^{1}H NMR of compound **2.**
7- Figure S2.2. ^{13}C NMR of compound **2.**
8- Figure S2.3. DEPT-135 of compound **2.**
9- Figure S2.4. HSQC of compound **2.**
10- Figure S2.5. HMBC of compound **2.**

Compound 1 NMR data:

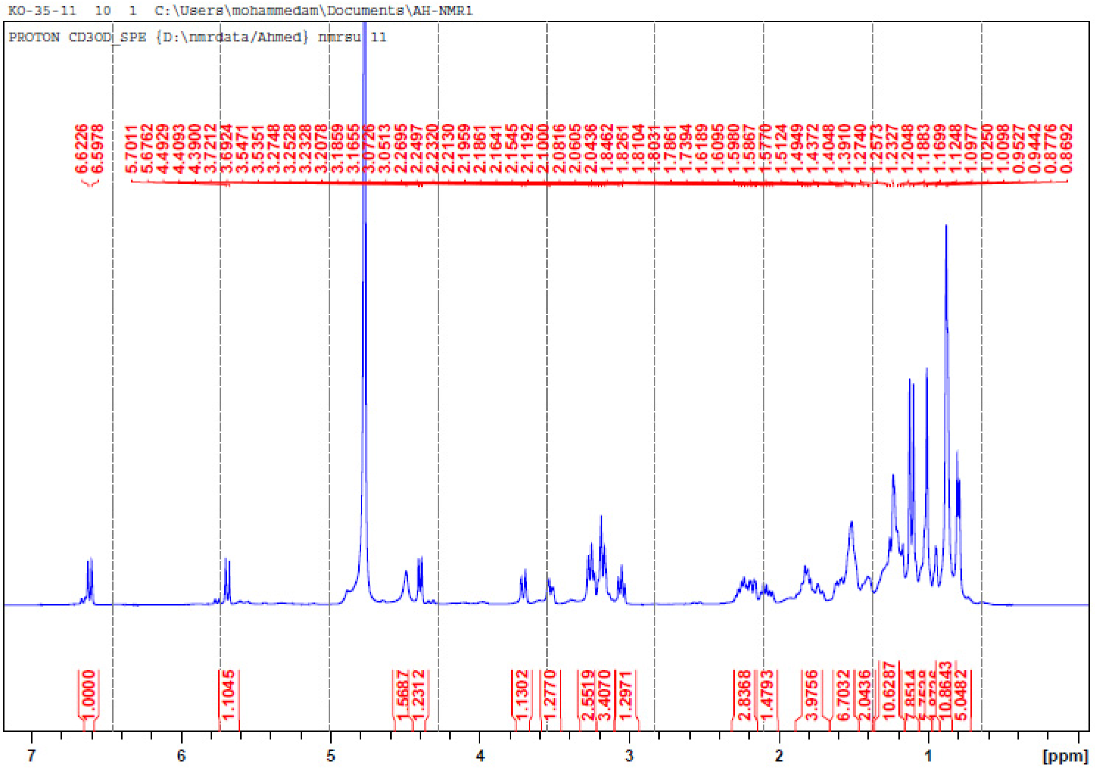

Figure S1.1. ^{1}H NMR of compound 1

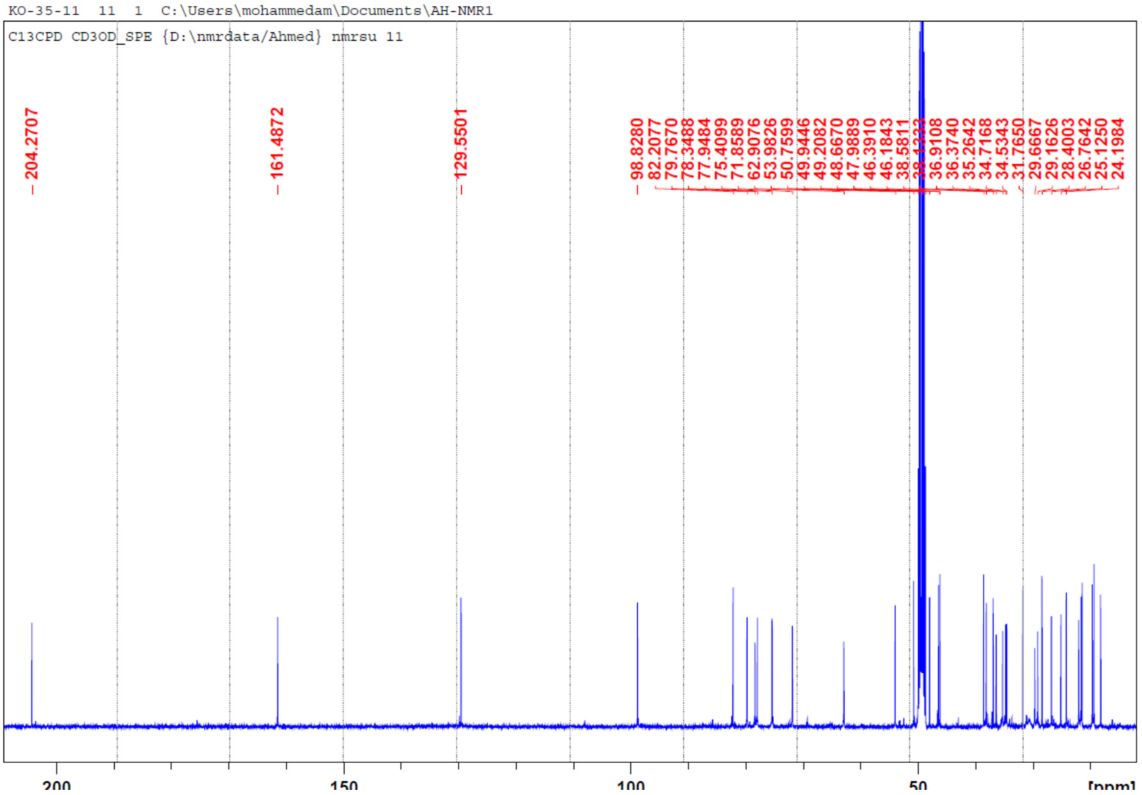

Figure S1.2. ^{13}C NMR of compound 1

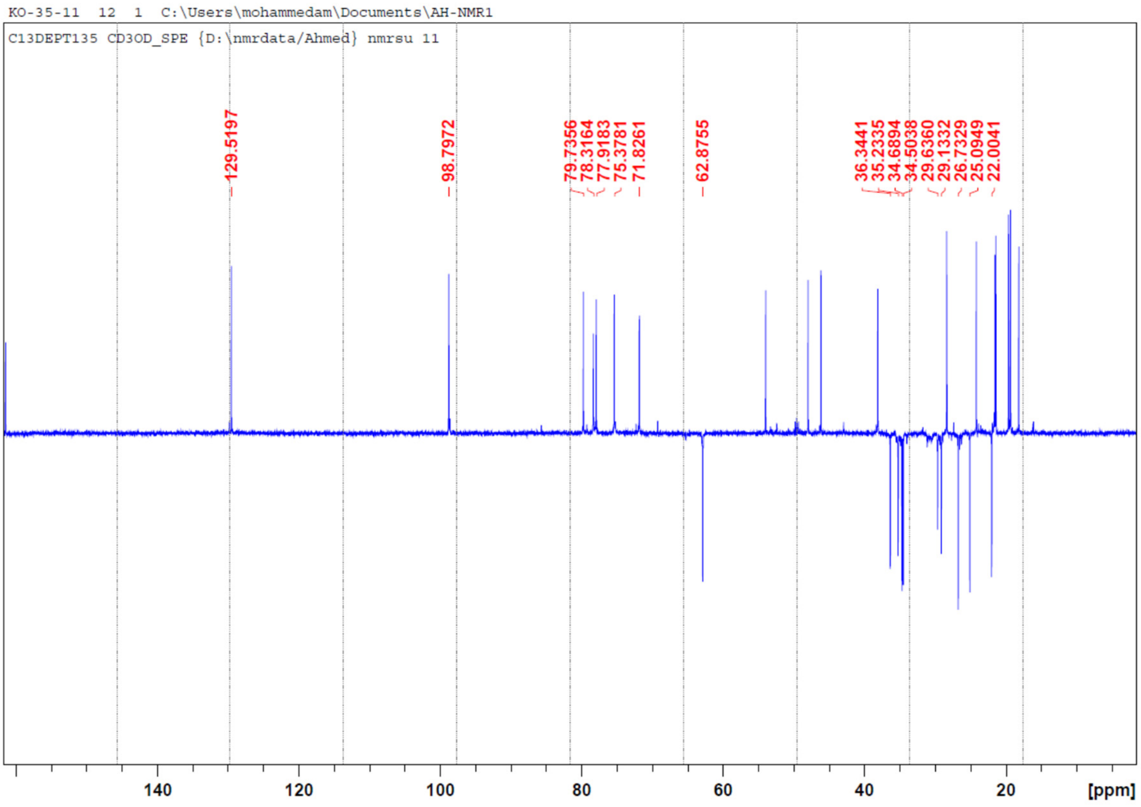

Figure S1.3. DEPT-135 of compound 1

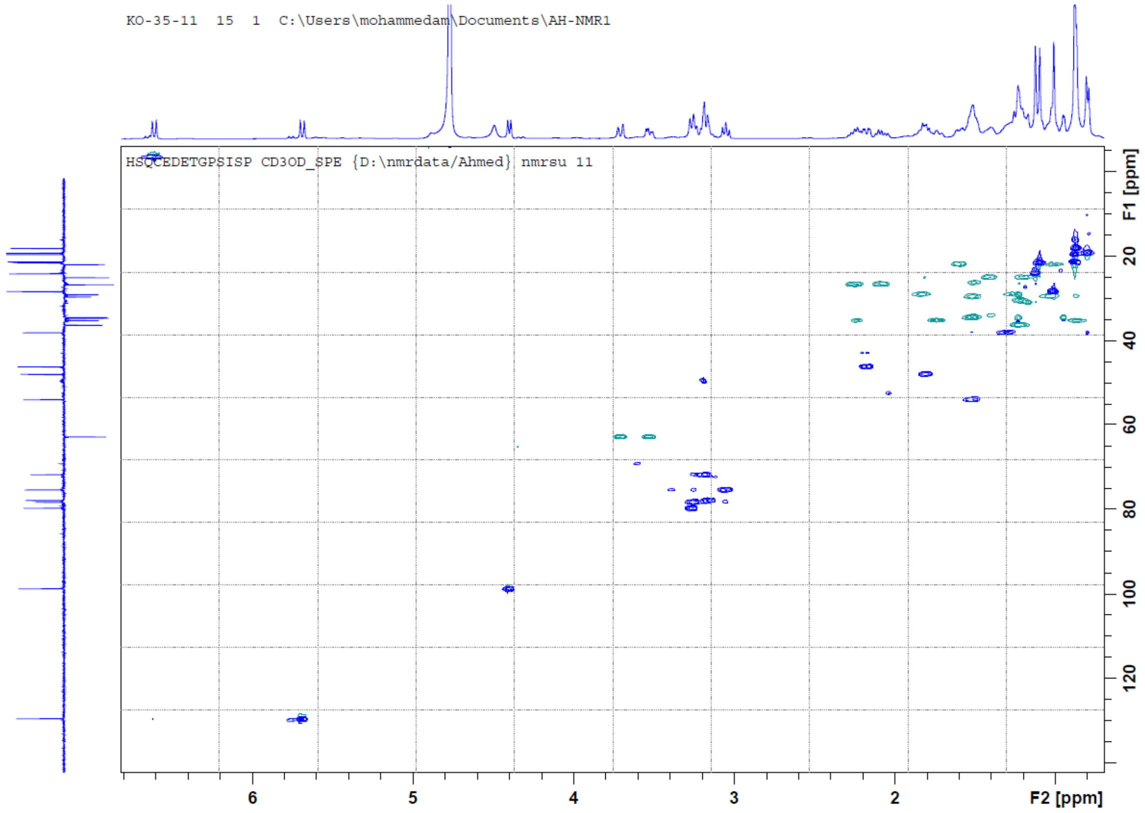

Figure S1.4. HSQC of compound **1**

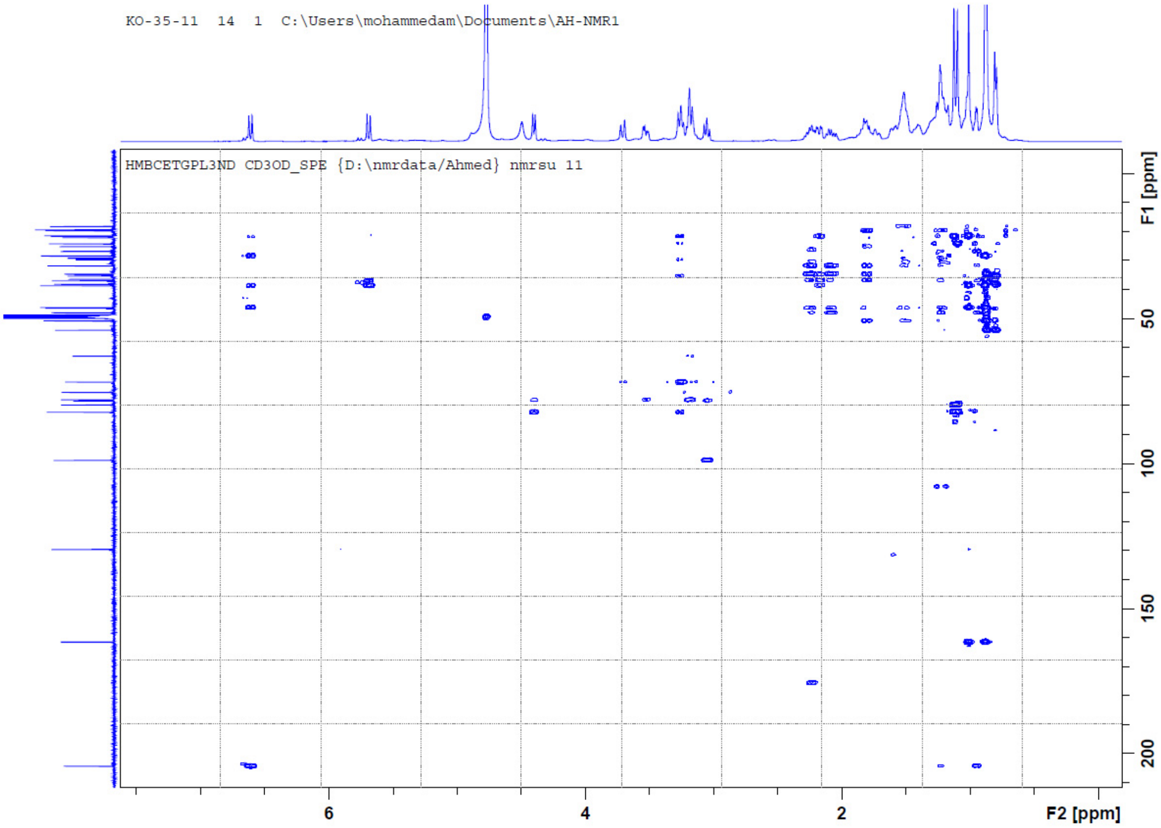

Figure S1.5. HMBC of compound **1**

Compound 2 NMR data:

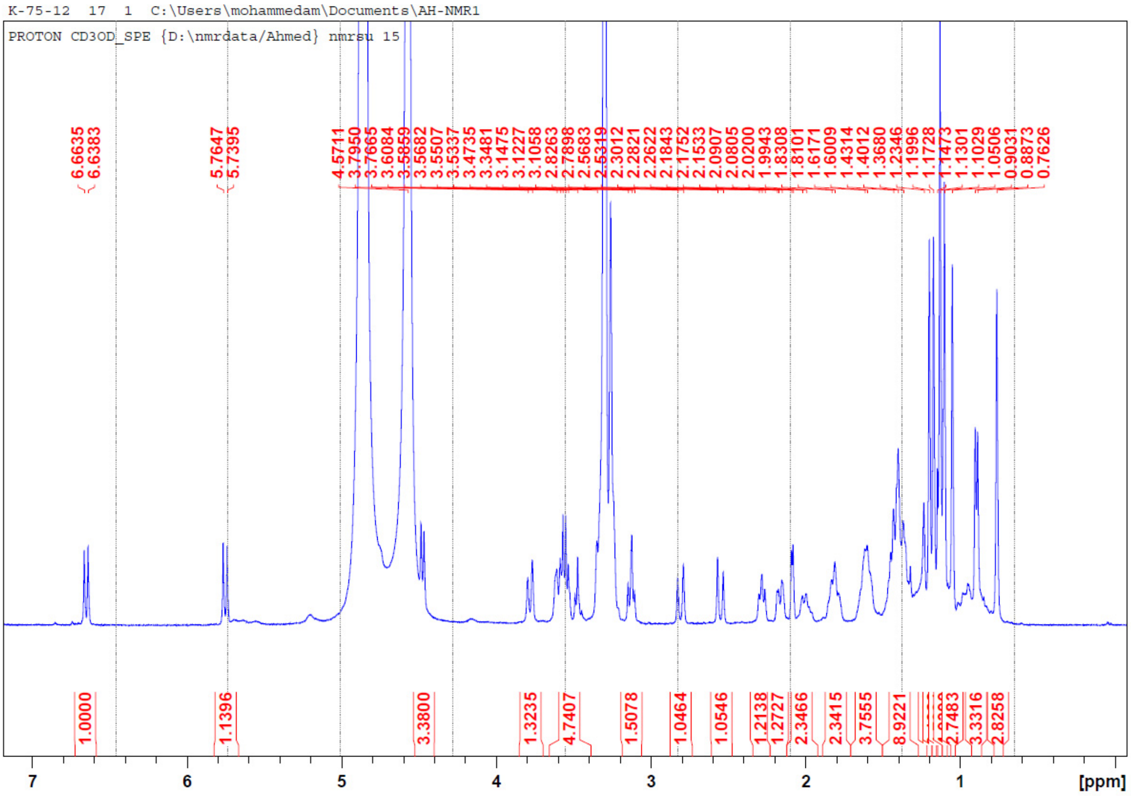

Figure S2.1. ^{1}H NMR of compound **2**

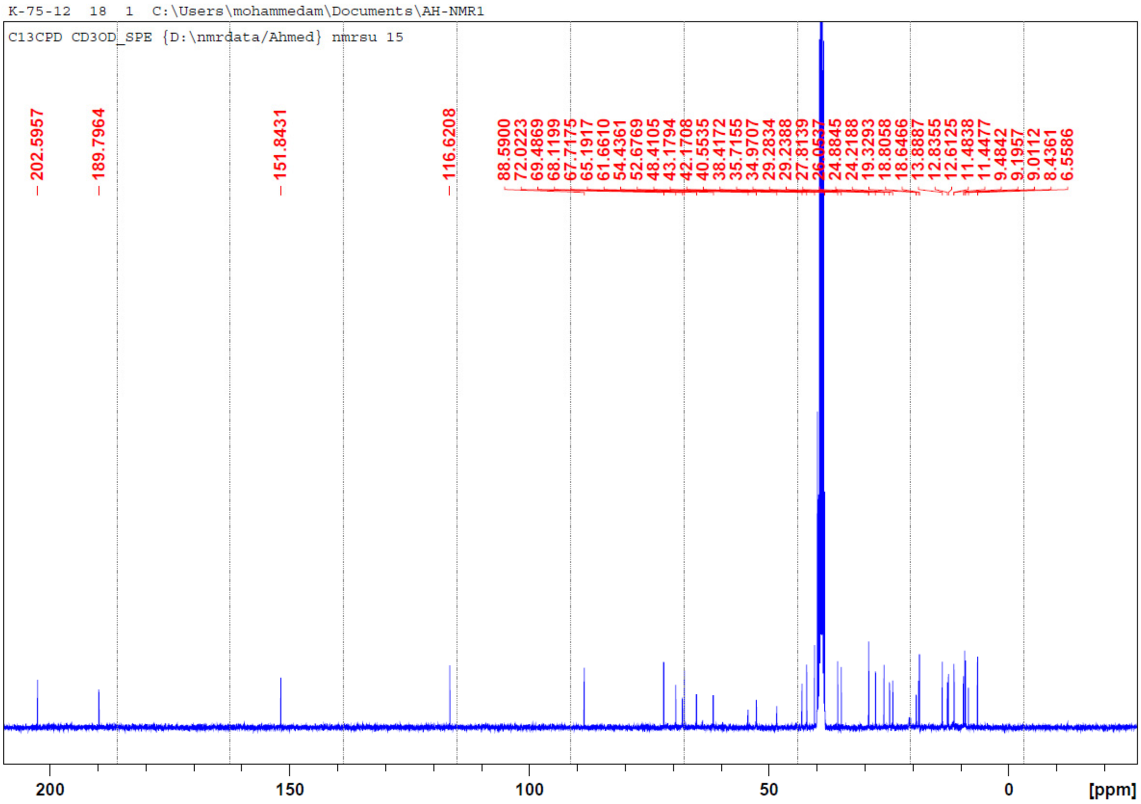

Figure S2.2. ^{13}C NMR of compound **2**

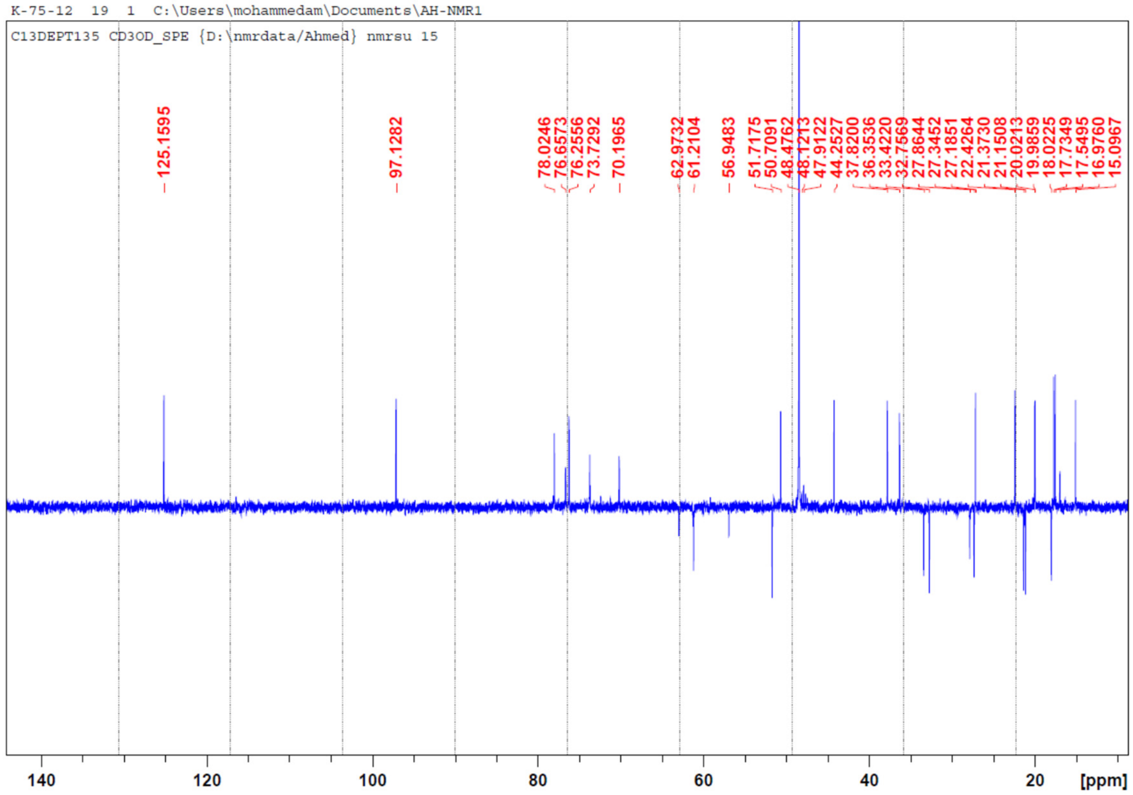

Figure S2.3. DEPT-135 of compound **2**

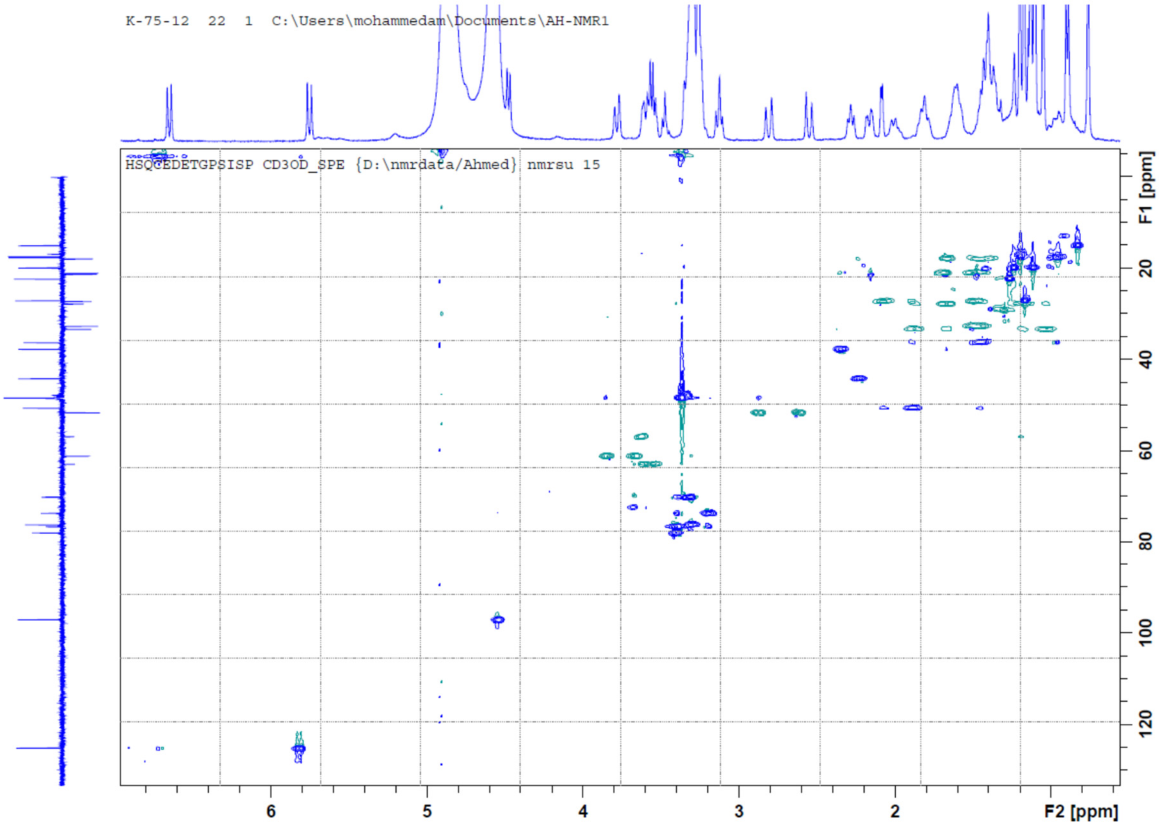

Figure S2.4. HSQC of compound **2**

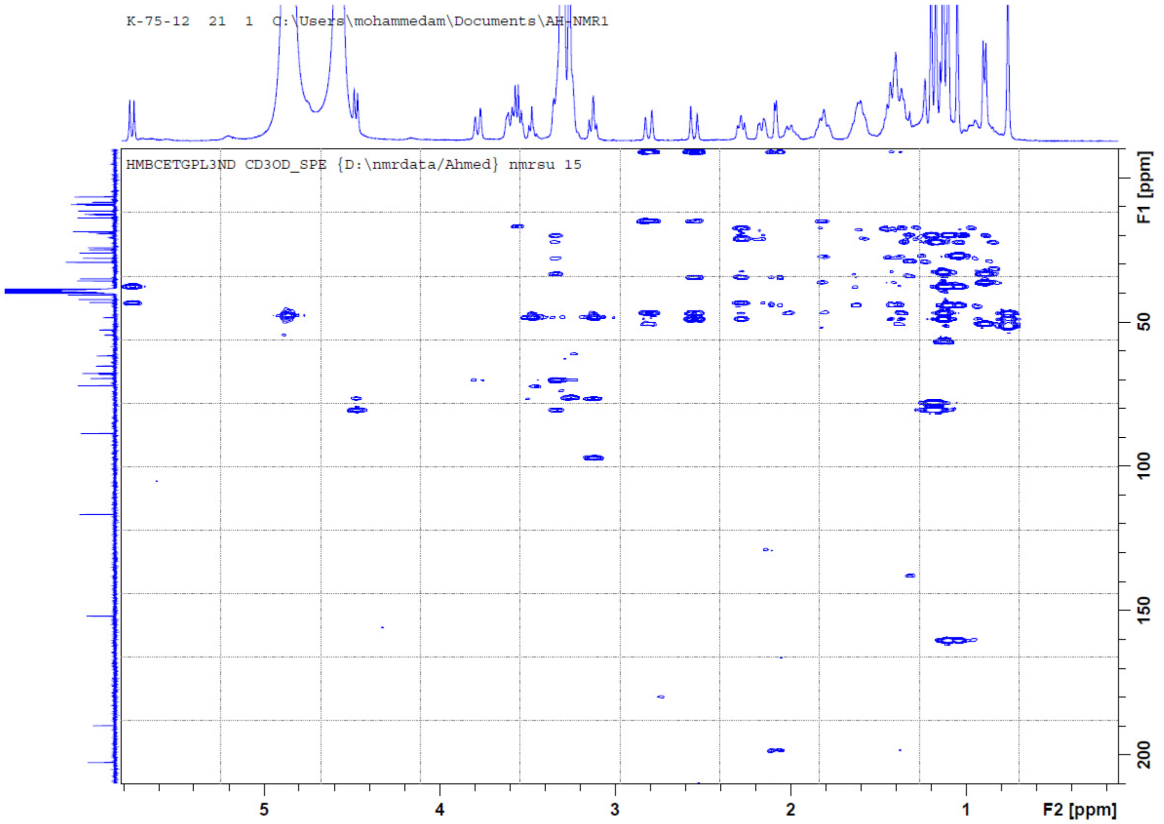

Figure S2.5. HMBC of compound **2**
